# ﻿The genus *Errastunus* in the Nearctic region (Hemiptera, Cicadellidae, Deltocephalinae)

**DOI:** 10.3897/zookeys.1178.105566

**Published:** 2023-09-05

**Authors:** Joel H. Kits

**Affiliations:** 1 Canadian National Collection of Insects, Arachnids, and Nematodes, Agriculture and Agri-Food Canada, Ottawa, ON, Canada Canadian National Collection of Insects Ottawa Canada

**Keywords:** Cytochrome oxidase I, DNA barcodes, introgression, leafhopper, Paralimnini, taxonomy

## Abstract

The leafhopper genus *Errastunus* contains grass-feeding leafhoppers in the deltocephaline tribe Paralimnini. The taxonomy of the genus in the Nearctic region has long been confused, with one to three distinct species recognized by different authors. Some populations have also been suggested to be adventive from Europe. Morphological and molecular data show that there are two distinct species in North America. These taxa are readily distinguishable morphologically although there is evidence of mitochondrial introgression between the species. The distribution of the two species based on historical material in collections suggests that *Errastunussobrinus* (DeLong & Sleesman, 1929) is native to North America, while *E.ocellaris* (Fallén, 1806) includes both native and adventive populations. A lectotype is designated for *E.sobrinus* and *Cicadaocellata* Scopoli, 1763 is established as a *nomen oblitum* with *Cicadaocellaris* as a *nomen protectum*.

## ﻿Introduction

**Figure 1.**
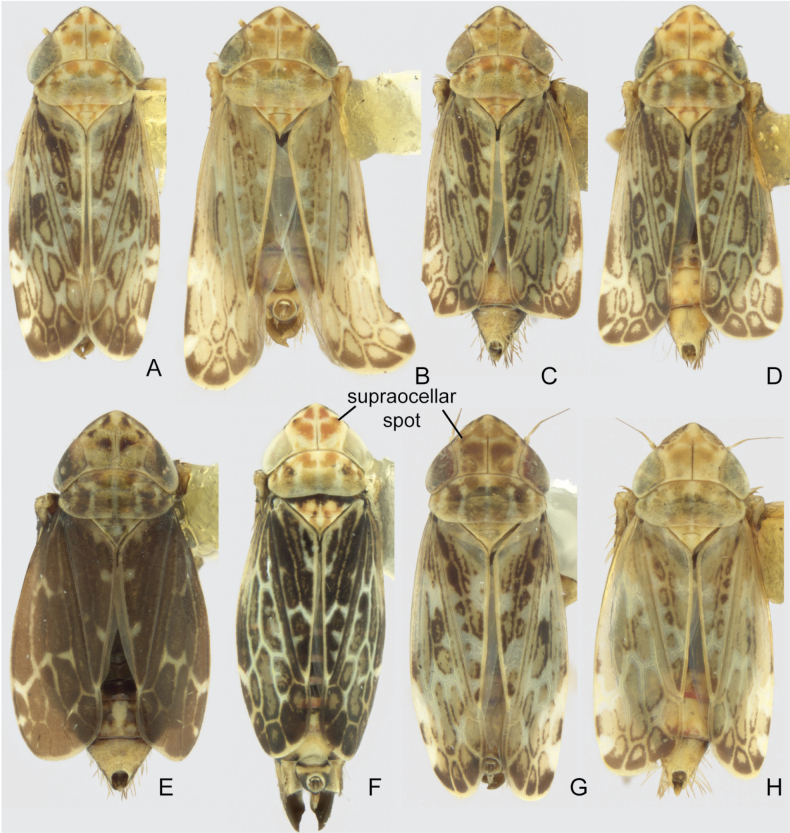
*Errastunus* dorsal habitus **A–F***Errastunusocellaris***A** CNC1317086 (Chatterton, ON) **B** CNC1317151 (Igls, Austria, 900 m) **C** CNC1317160 (Oxford, England) **D** CNC1317521 (Obergurgl, Austria, 1950 m) **E** CNC1317524 (Obergurgl, Austria, 1950 m) **F** CNC1615789 (Richardson Mtns, YT, note the extended abdomen is an artifact of preservation) **G, H***Errastunussobrinus***G**CNC#HEM403281 (Parc de la Gaspesie, QC) **H** CNC1317427 (Elkwater Park, AB).

**Figure 2.**
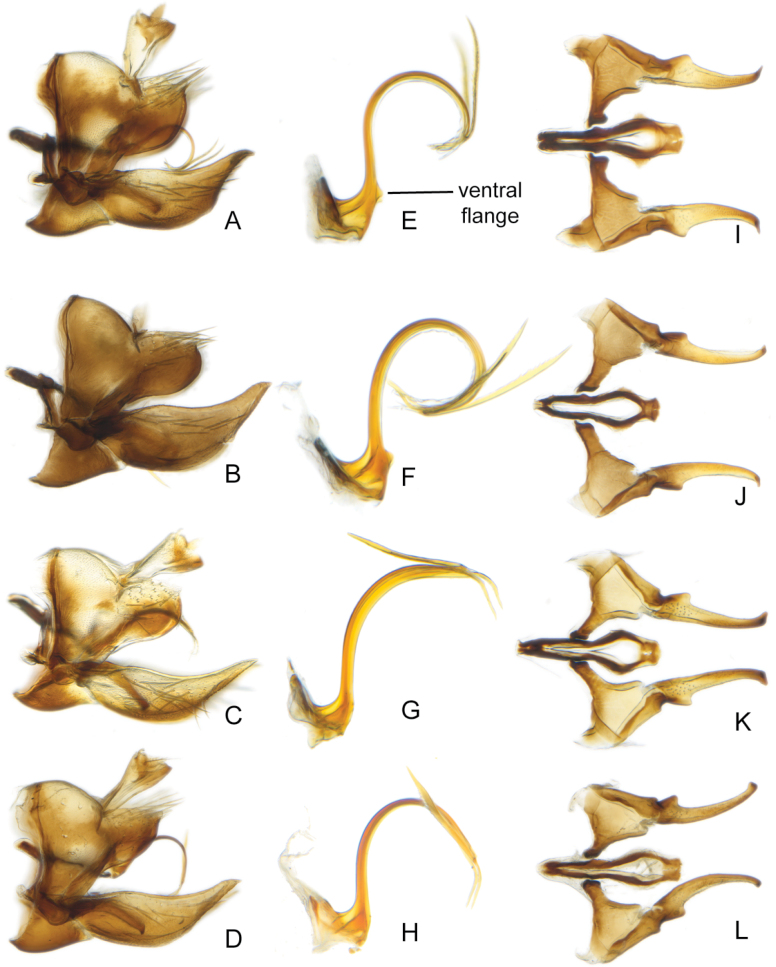
*Errastunusocellaris*, male genitalia **A–D** pygofer, lateral **E–H** aedeagus, lateral **I–L** connective and styles, dorsal **A, E, I** CNC1317192 (King Salmon, AK) **B, F, J** CNC1317520 (Obergurgl, Austria, 1950 m) **C, G, K** CNC661822 (Big Muddy, SK) **D, H, L** CNC1317183 (Leeds, England).

The leafhopper genus *Errastunus* Ribaut, found in Europe and North America, contains boldly marked, grass-feeding leafhoppers (Fig. 1) placed in the deltocephaline tribe Paralimnini based on the racket-shaped connective (Fig. 2). It appears to be closely related to *Adarrus* Ribaut and has sometimes been treated as synonymous with that genus; it can be distinguished based on the subapical, dorsal gonopore and additional crossveins in the clavus (Dmitriev 1999). Some North American authors have placed the constituent species in *Latalus* DeLong & Sleesman (e.g., DeLong and Sleesman 1929; Hamilton 1983); however, *Errastunus* differs from *Latalus* in having long, pointed subgenital plates with multiple rows of setae, no process on the pygofer, and an elongate median lobe of the style apophysis (Oman 1949). The type species, *E.ocellaris* (Fallén, 1806), is widespread in the Palearctic region and of uncertain status in the Nearctic region. Two additional taxa have been treated as either distinct species or as synonyms of *E.ocellaris* by various authors: *E.sobrinus* (DeLong & Sleesman, 1929) described from North America, and *E.ocellaristatraensis* (Heller, 1975) described from Slovakia. Dmitriev (2001) described a separate subgenus Anadarrus to contain E. (A.) daedaleus (Logvinenko), with the other species above placed in the nominate subgenus.

The identities of the species of *Errastunus* in the Nearctic region have been confused for many years. The earliest Nearctic record listed by Metcalf (1967) is from Osborn and Ball (1897) who recorded *E.ocellaris* from Colorado (not Iowa as indicated by Metcalf). Lawson (1922) later listed the species from Kansas, Osborn (1922) recorded it from New York, and Downes (1924) added a record from British Columbia. DeLong (1926) repeated these records and considered the species to occur in both Europe and North America. DeLong and Sleesman (1929) later considered Nearctic populations to be distinct from those in the Palearctic, based on differences in body form and the female 7^th^ sternite. They described these as a variety under the name Latalusocellarisvar.sobrinus, but stated it “is in all probability a separate species.” Only material from Slave Lake, Alberta, and “northern New York State” was explicitly included in this description although the authors imply that this is the only form present in the Nearctic.

Walley (1932) agreed with DeLong and Sleesman that the Nearctic form was specifically distinct from those in Europe, and recorded it (as *Deltocephalussobrinus*) from Quebec, Manitoba and British Columbia. Medler (1943) recorded *E.sobrinus* (and not *E.ocellaris*) from Minnesota.

Other authors continued to record *E.ocellaris* from North America, such as DeLong and Knull (1945) who repeated DeLong’s (1926) records of this species but also recorded *E.sobrinus* from Alberta. Oman (1949) listed *E.ocellaris* from the northeastern, central, and northwestern regions of the Nearctic, and *E.sobrinus* from Alberta. Strickland (1953) recorded both species from Alberta, based on determinations by Bryan Beirne (then at the Canadian National Collection). There are specimens in the Canadian National Collection identified as *E.sobrinus* by Beirne in 1950–1951, although Beirne (1956) later treated *E.sobrinus* as a junior synonym of *E.ocellaris*.

Hamilton (1983) later reviewed the status of these taxa (under the genus *Latalus*), treating *E.ocellaris* and *E.sobrinus* as distinct species, both present in the Nearctic. He regarded *E.sobrinus* as a native Nearctic species occurring in the boreal zone, while *E.ocellaris* was treated as a Palearctic species introduced into eastern Canada and the adjacent United States. Later, Hamilton (1997) recorded *E.tatraensis* (as a full species) from northwestern North America in Alaska, Yukon, and Northwest Territories. However, characters separating these taxa have not been clearly described and some authors (e.g., Dmitriev et al. 2022) currently consider *E.sobrinus* and *E.tatraensis* as synonyms of *E.ocellaris*. Here I review the status of the taxa present in the Nearctic, provide characters for their separation and detail their distribution.

## ﻿Material and method

Specimens examined (see Suppl. material 1) and types are from the following collections, with abbreviations:

**BIOUG**Biodiversity Institute of Ontario (Guelph, Ontario, Canada);

**CNC**Canadian National Collection of Insects, Arachnids, and Nematodes (Ottawa, Ontario, Canada);

**INHS**Illinois Natural History Survey (Champaign, Illinois, United States);

**OSUC**C. A. Triplehorn Insect Collection, The Ohio State University (Columbus, Ohio, United States);

**UNHC**University of New Hampshire Insect Collection (Durham, New Hampshire, United States);

**USNM**Smithsonian National Museum of Natural History (Washington, D.C., United States).

Images were taken using a Leica M205C stereomicroscope with 1.6× objective, stacked using Zerene Stacker (Zerene Systems, Richland, WA, USA), edited using Adobe Photoshop CS6, and assembled into plates using Adobe Illustrator CS6 (Adobe Inc., San Jose, CA, USA). Maps were created using QGIS v.3.20.0 (QGIS.org). Synonymies presented here are not complete and emphasize significant nomenclatural changes since Metcalf (1967).

Published COI sequences for specimens held in the CNC (Foottit et al. 2014; Gwiazdowski et al. 2015) and BIOUG (Hebert et al. 2016) were combined with additional COI sequences generated for this project (Table 1). For new sequences, DNA was extracted from single legs of specimens using 10 uL of QuickExtract buffer (Lucigen, Middleton, WI, USA), incubated at 56 °C overnight followed by 95 °C for 5 mins. A 418 bp fragment of COI (corresponding to the 3’ end of the standard DNA barcode region) was amplified using the primers BF3 (5’-CCHGAYATRGCHTTYCCHCG; Elbrecht et al. 2019) and C_LepFolR (5’-TAAACTTCWGGRTGWCCAAAAAATCA; Hernández-Triana et al. 2014) with 9 bp index tags attached. PCRs were carried out with 15 uL reaction volumes with 0.3 units KAPA2G Robust polymerase (KAPA Biosystems, Cape Town, South Africa), 0.33 µM of each primer, 2 mM MgCl_2_, and 0.2 mM dNTPs in 1X KAPA buffer B, with 1 µL of template. Cycling conditions were 95 °C × 180 s; 5 cycles of 95 °C × 15 s, 45 °C × 15 s, 72 °C × 30 s; 35 cycles of 95 °C × 15 s, 51 °C × 15 s, 72 °C × 30 s; 72 °C × 60 s. PCRs were carried out on 96-well plates (with material for unrelated projects), and each specimen had a unique combination of tags.

**Table 1.** Data for sequenced specimens.

Specimen ID	GenBank Accession	BOLD Process ID	Taxon	Locality	Depository
BIOUG24070-C06	MG404290	SSKNA3723-15	*Errastunusocellaris*	CAN: BC	BIOUG
BIOUG24070-F08	MG406968	SSKNA5866-15	*Errastunusocellaris*	CAN: BC	BIOUG
BIOUG24070-H01	MG399140	SSKNA7962-15	*Errastunusocellaris*	CAN: BC	BIOUG
BIOUG24414-A10	MG397261	SSKNA9802-15	*Errastunusocellaris*	CAN: BC	BIOUG
CNC#HEM305694	OQ685764	AHCNC606-13	*Errastunusocellaris*	USA: NH	UNHC
CNC#HEM403279	OQ685762	CNCHF852-12	*Errastunusocellaris*	CAN: ON	CNC
CNC#HEM403280	OQ685763	CNCHF853-12	*Errastunusocellaris*	CAN: YT	CNC
CNC#HEM403283	OQ685765	CNCHF856-12	*Errastunusocellaris*	CAN: YT	CNC
CNC#HEM403284	OQ685768	CNCHF857-12	*Errastunusocellaris*	CAN: YT	CNC
CNC1316991	OQ649783		*Errastunusocellaris*	USA: NH	CNC
CNC1317007	OQ649784		*Errastunusocellaris*	USA: NH	CNC
CNC1317100	OQ649785		*Errastunusocellaris*	USA: NY	CNC
CNC1317150	OQ649781		*Errastunusocellaris*	Austria	CNC
CNC1317158	OQ649782		*Errastunusocellaris*	United Kingdom	CNC
CNC1317520	OQ649788		*Errastunusocellaris*	Austria	CNC
CNC1317525	OQ649789		*Errastunusocellaris*	Austria	CNC
CNC1615748	OQ649790		*Errastunusocellaris*	CAN: YT	CNC
CNC1615798	OQ649791		*Errastunusocellaris*	CAN: YT	CNC
CNC661822	OQ649792		*Errastunusocellaris*	CAN: SK	CNC
BIOUG06007-B07	KR561122	SSEIB4991-13	*Errastunussobrinus*	CAN: AB	BIOUG
BIOUG08388-A02	KR561203	SSPAC8479-13	*Errastunussobrinus*	CAN: SK	BIOUG
BIOUG08388-D08	KR578594	SSPAC8215-13	*Errastunussobrinus*	CAN: SK	BIOUG
BIOUG08388-E07	KR560380	SSPAC8226-13	*Errastunussobrinus*	CAN: SK	BIOUG
BIOUG08464-G05	KR562589	SSPAC9906-13	*Errastunussobrinus*	CAN: SK	BIOUG
BIOUG08503-A11	KR577339	SSPAC10328-13	*Errastunussobrinus*	CAN: SK	BIOUG
BIOUG09175-C05	KR581470	SSPAC12902-13	*Errastunussobrinus*	CAN: SK	BIOUG
BIOUG09180-H03	KR565466	SSPAC13435-13	*Errastunussobrinus*	CAN: SK	BIOUG
CNC#HEM403281	OQ685767	CNCHF854-12	*Errastunussobrinus*	CAN: QC	CNC
CNC#HEM403282	OQ685766	CNCHF855-12	*Errastunussobrinus*	USA: AK	CNC
CNC1317360	OQ649786		*Errastunussobrinus*	USA: CO	CNC
CNC1317518	OQ649787		*Errastunussobrinus*	CAN: AB	CNC
CNC#HEM403285	KR036843	CNCHF858-12	*Lataluscurtus*	CAN: BC	CNC
CNC#HEM403303	KR038259	CNCHF876-12	*Latalushistrionicus*	USA: AZ	CNC

Libraries were then prepared for pooled PCR products from each plate using a PCR-free protocol modified from standard Illumina library preparation protocols (Meyer and Kircher 2010). Pooled amplicons were cleaned using SPRI beads and 1 µg used for library preparation. Pools were phosphorylated with T4 polynucleotide kinase (1 µL in 50 µL 1× T4 ligase buffer), A-tailed with Taq polymerase (1 µL in 50 uL 1× Taq buffer, with 0.2 mM dATP and 1.5 mM MgCl_2_), and Lucigen NxSeq adaptors (Lucigen, Middleton, WI, USA) added with T4 DNA ligase (2.5 µL in 50 µL 1× T4 ligase buffer, with 10% PEG-8000 and 2.5 µL adaptor) (all enzymes and buffers NEB Canada, Whitby, ON). Each plate was tagged with a unique i7 index. Libraries were cleaned up between each step using SPRI beads or PEG-NaCl solution (Fisher et al. 2011).

Libraries were sequenced on the Illumina MiSeq platform (2 × 300 bp reads). Resulting fastq files were demultiplexed and trimmed with CutAdapt (Martin 2011), unique sequence variants identified with dada2 (Callahan et al. 2016), and the most abundant variant retained for each sample.

Sequences were analysed using MEGA11 (Tamura et al. 2021). Sequences were initially aligned using the Muscle algorithm (Edgar 2004) and then refined by eye. The neighbour-joining trees and distance metrics were both produced using p-distances. Sequences from two species of the related genus *Latalus* were used as outgroups.

## ﻿Results

**Figure 3.**
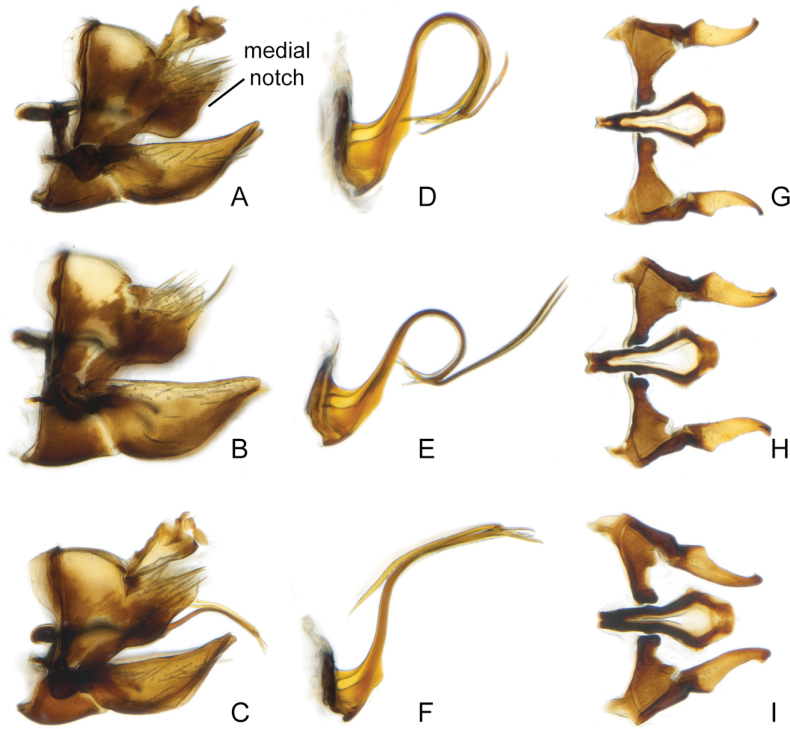
*Errastunussobrinus*, male genitalia **A–C** pygofer, lateral **D–F** aedeagus, lateral **G–I** connective and styles, dorsal **A, D, G** CNC1317444 (Atlin, BC) **B, E, H** CNC1317472 (Waskaganish, QC) **C, F, I** CNC1317518 (Waterton Lakes, AB).

Based on morphology, specimens of *Errastunus* from North America can be divided into two groups, which correspond to *E.sobrinus* and *E.ocellaris*. In males, there is a consistent and obvious difference in the shape of the pygofer lobe which is entirely convex along the postero-ventral margin in *E.ocellaris* and indented in *E.sobrinus*. There are also less distinct differences in the length of the subgenital plates and shape of the flange at the base of the aedeagal shaft (Figs 2, 3), as detailed in the below key. In addition, the style apophysis is typically shorter and the posterior part of the connective proportionally broader and more evenly rounded in *E.sobrinus*, although these differences are subtle and difficult to use for identification. The only previously published character suggested to separate males is longer aedeagal processes in *E.sobrinus* (Hamilton 1983). This presumably refers to the longer dorsal rather than the inconspicuous ventral processes, but both vary considerably in both species with extensive overlap and are not useful for separation.

**Figure 4.**
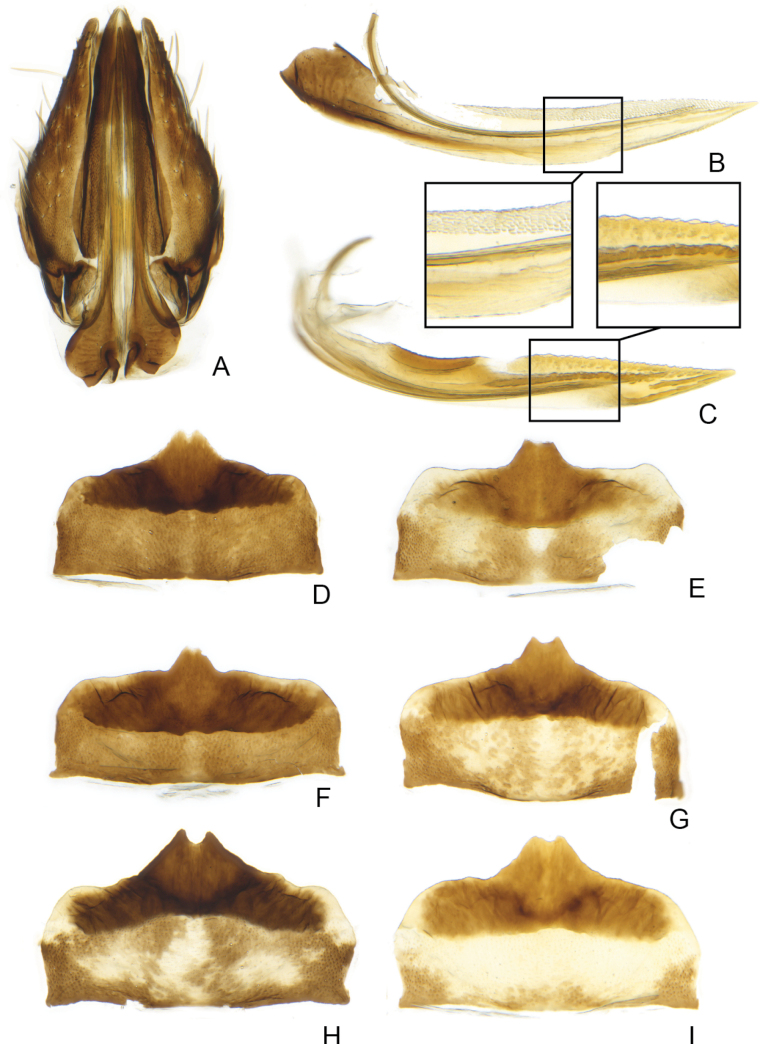
*Errastunusocellaris*, female genitalia **A** genital capsule, ventral **B** first valvula, with enlargement **C** second valvula, with enlargement **D–I** sternite 7, ventral **A–D** CNC1317157 (Oxford, England) **E** CNC1317039 (Brockville, ON) **F** CNC1316995 (Mt. Washington, NH) **G** CNC1317204 (King Salmon, AK) **H** CNC1615794 (Richardson Mtns, YT) **I** CNC1317205 (King Salmon, AK).

**Figure 5.**
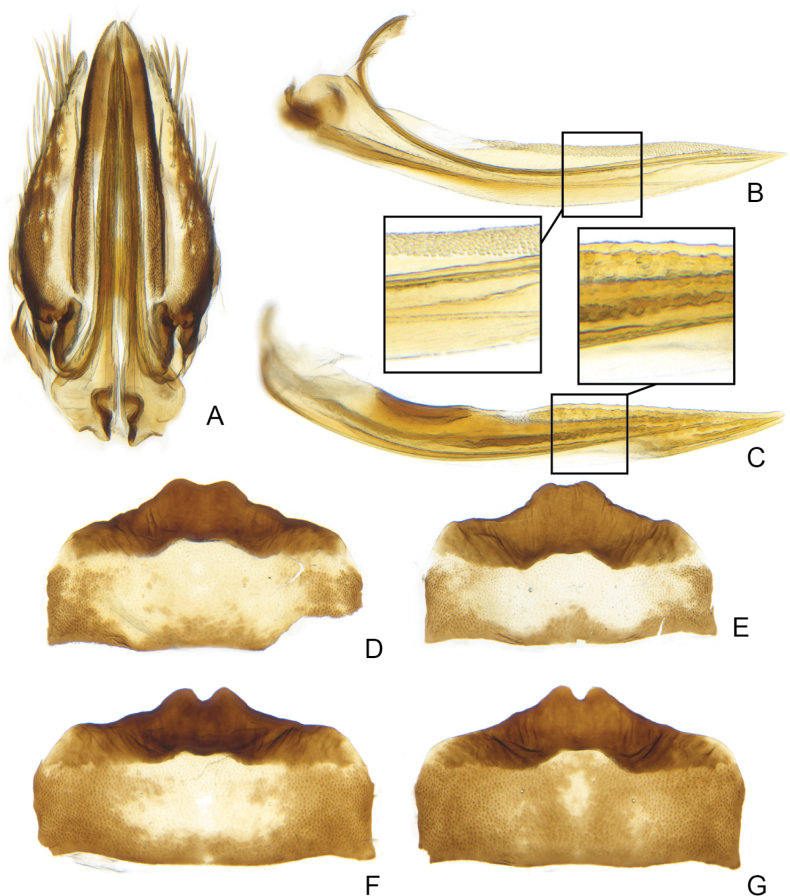
*Errastunussobrinus*, female genitalia **A** genital capsule, ventral **B** first valvula **C** second valvula **D, E** sternite 7, ventral **A–D** CNC1317465 (Waskaganish, QC) **E** CNC1317360 (La Plata Co., CO) **F** CNC1317260 (Whitefox, SK) **G** CNC1317270 (Rycroft, AB).

**Figure 6.**
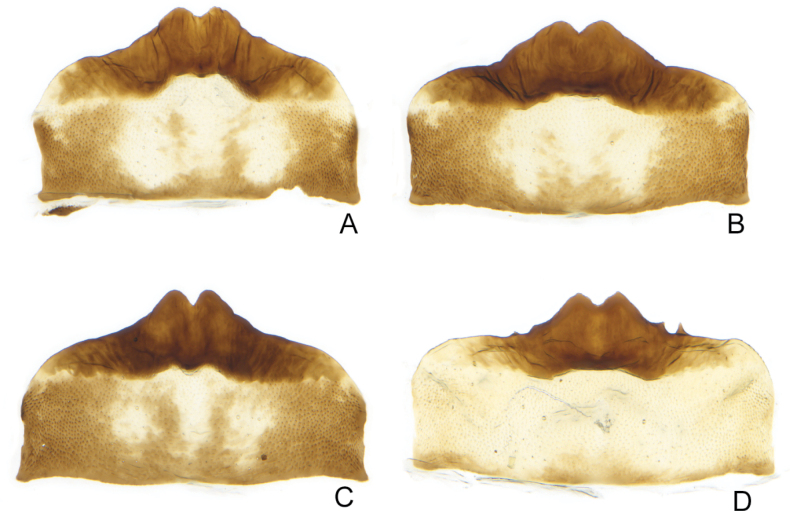
*Errastunus* females from Alaska and Yukon, sternite 7 **A–C** presumed *E.sobrinus***D** presumed *E.ocellaris***A** CNC1317218 (Skagway, AK) **B** CNC1317220 (Circle Hot Springs, AK) **C** CNC1317219 (Matanuksa, AK) **D** CNC1317217 (Carmacks, YT).

The first character suggested to separate the species was the shape of the female 7^th^ sternite (DeLong and Sleesman 1929), with *E.sobrinus* having a broad projection with rounded teeth and *E.ocellaris* having a narrower projection with pointed teeth. These character states are usually clearly distinct and readily separate most females of the two species (Figs 4, 5). However, there is some variation among both species in this character and a few unassociated females from areas where both species may occur could only be identified with low confidence (Fig. 6).

**Figure 7.**
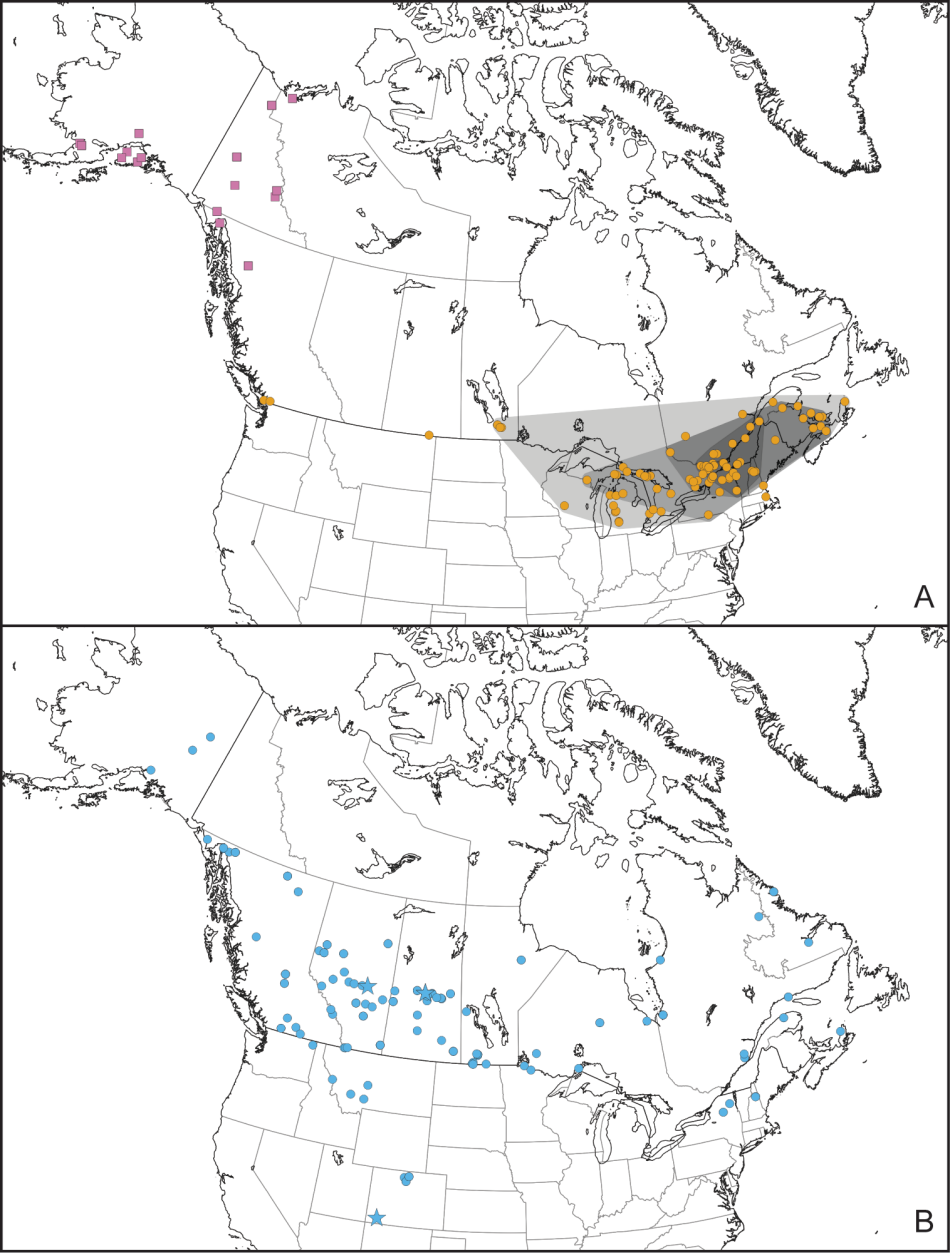
Distribution of *Errastunus* in North America **A***E.ocellaris*, adventive (orange circles) and native (purple squares) populations. Shaded areas outline cumulative distribution of the eastern population by decade, from 1960 (darkest) to 1990 (lightest) **B***E.sobrinus* (blue circles). Localities for specimens with introgressed COI indicated by stars.

Specimen data suggests distinct distributions for the two species, with only partial overlap (Fig. 7). *Errastunussobrinus* has a boreo-montane distribution, occurring across Canada and the adjacent United States, south in the Rocky Mountains to Colorado.

Records for *E.ocellaris* are clustered in the east, primarily from the Great Lakes to the Atlantic coast, and in the northwest including Alaska, Yukon, and adjacent parts of the Northwest Territories and British Columbia. There are a few records from the southern Prairie provinces and near Vancouver. There is a clear signal of range expansion in the east (Fig. 7A). The earliest records are clustered in southern Quebec, eastern Ontario, and northern New York, with later records gradually spreading from this area.

Eastern North American *E.ocellaris* are not separable morphologically from European specimens, while northwestern specimens differ only slightly in the shape of the style and length of the subgenital plates, and are quite similar to higher elevation European specimens.

**Figure 8.**
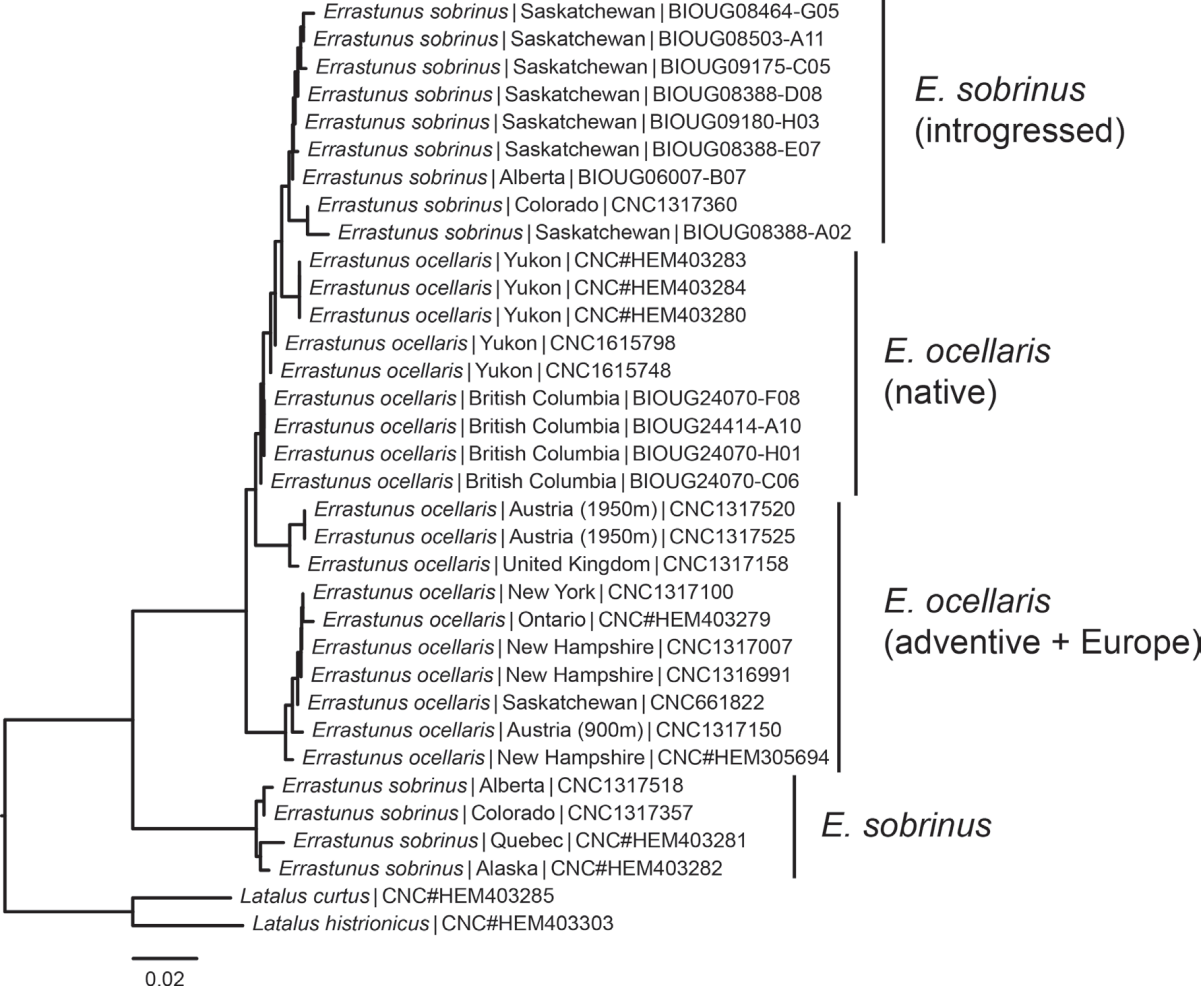
Neighbour-joining tree of COI sequences from *Errastunus* specimens. Tip labels indicate the taxon (based on morphological characters), collection locality, and specimen identifier.

Newly sequenced and previously published DNA barcodes for five specimens of *E.sobrinus*, five specimens of northwestern *E.ocellaris*, six specimens of eastern *E.ocellaris*, and four specimens of European *E.ocellaris*, all from specimens in the CNC, were analysed. These initial sequences were divided into two distinct clusters, largely matching the morphological species concept. However, a single specimen identified morphologically as *E.sobrinus* fell within the cluster containing *E.ocellaris* specimens, clustering near sequenced specimens from Yukon. Based on this, additional specimens from BIOUG which had previously been sequenced and fell in the same cluster were examined; a number of these were morphologically identified as *E.sobrinus*. Analysis of sequences from all morphologically identified specimens (Fig. 8) show two clusters separated by a mean p-distance of 9.18% (mean distance within clusters 0.86–1.82%).

### ﻿ Errastunus (Errastunus)

Taxon classificationAnimaliaHemipteraCicadellidae

Ribaut, 1946

EF0491A5-0F0C-5024-AEC2-400C3619EBDC

Errastunus Ribaut, 1946: 83 (new genus).
Adarrus (Errastunus) — Emeljanov 1966 (as subgenus).
Latalus (Errastunus) — Hamilton 1983 (as subgenus).
Errastunus (Errastunus) — Dmitriev 2001 (as subgenus).

#### Type species.

*Cicadaocellaris* Fallén, 1806 (by original designation).

#### Diagnosis.

Distinguished from other Paralimnini by the following combination of characters: male plates with multiple, uneven marginal rows of macrosetae, apices elongate and pointed; aedeagus symmetrical with dorso-apical gonopore and two pairs of subapical appendages; clavus with additional crossveins.

### ﻿Key to *Errastunus* species of the Nearctic region

**Table:** 

1	Male pygofer lobe with entirely convex postero-ventral margin (Fig. 2A–D). Male plates usually extending well beyond pygofer apex, plates over 1.33X (Fig. 2A) and often 1.5X pygofer length (Fig. 2D). Aedeagus with ventral flange near base of shaft absent (Fig. 2G, H) or subtriangular, about as long as wide (Fig. 2E, F). Female 7^th^ sternite with medial projection bearing two sharply pointed teeth, the projection usually narrow (Fig. 4D–I). Spots on head and pronotum usually orange, usually with a dark brown supraocellar spot (Fig. 1A–F)	***E.ocellaris***
–	Male pygofer lobe with medial notch in postero-ventral margin (Fig. 3A–C). Male plates extending only slightly beyond pygofer apex, plates less than 1.33X pygofer length (Fig. 3A–C). Aedeagus with rounded ventral flange near base, flange usually much longer than wide (Fig. 3D–F). Female 7^th^ sternite with a broad medial projection bearing two rounded teeth (Fig. 5D–G). Spots on head and pronotum usually yellow to brown, supraocellar spot concolourous with other spots (Fig. 1G, H)	***E.sobrinus***

### ﻿ Errastunus ocellaris

Taxon classificationAnimaliaHemipteraCicadellidae

(Fallén)

83ED0324-C0BE-5618-9CAC-3106782466C7

Figs 1A–F , 2 , 4

Cicada ocellata Scopoli, 1763: 116 (*nomen oblitum*).
Cicada ocellaris Fallén, 1806: 20. Type locality: Scania, Sweden (*nomen protectum*).
=Jassus (Deltocephalus) notatifrons Kirschbaum, 1868: 141 (syn. Wagner 1939).
=Deltocephalussachalinensis Matsumura, 1915: 168 (syn. Vilbaste 1969).
Latalus ocellaris (Fallén) — DeLong and Sleesman 1929 (comb. nov.).
Errastunus ocellaris (Fallén) — Ribaut 1946 (comb. nov.).
Adarrus (Errastunus) ocellaris (Fallén) — Emeljanov 1966 (comb. nov.).
Adarrus ocellaris tatraensis Heller 1975 (new subspecies).
Latalus (Adarrus) tatraensis (Heller) — Hamilton 1997 (revised status).

#### Material examined.

368 specimens (see Suppl. material 1).

#### Distribution.

Widespread in the Palearctic region, from western Europe and northern Africa to Korea and the Russian Far East (Nast 1972). In the Nearctic region, occurs in the northwest (Alaska, Yukon, Northwestern Territories and northern British Columbia) and across southern Canada and the northern United States, with records concentrated in the east.

#### Host plants.

Feeds on a variety of cool season grasses. Nickel (2003) reports grasses including *Dactylisglomerata*, *Elymusrepens*, *Calamagrostis* spp., and *Holcus* spp. as hosts in central Europe. Recorded hosts for Nearctic specimens are *Elymustrachycaulus* (Bourget, ON), *Bromus* sp. (Oxbow, MI and Gravel Lake, YT), *Calamagrostiscanadensis* (Richardson Mountains, YT), and *Calamagrostis* sp. (King Salmon, AK and Aho Lake, AK).

#### Remarks.

The oldest name for this species, *Cicadaocellata* Scopoli, 1763, is a *nomen oblitum* as it has not been used as a valid name after 1899 (Article 23.9.1.1 of the International Code of Zoological Nomenclature (ICZN 1999)). To my knowledge, the most recent use of this name other than those excluded under Article 23.9.6 is Claus (1884, p. 892). *Cicadaocellaris* Fallén, 1806 is a *nomen protectum* based on the works listed in the Appendix 1 which fulfill the criteria of Article 23.9.1.2. The valid name of this species is thus *Cicadaocellaris* Fallén in accordance with Article 23.9.2.

Hamilton (1997) treated the northwestern populations of this species as a distinct species, Latalus (Adarrus) tatraensis (Heller). He linked these to high elevation populations from the Tatra Mountains in Slovakia which Heller (1975) had described as a subspecies. Heller distinguished his new subspecies based on dramatic colour differences, as well as slight differences in the male and female genitalia.

The status of the high elevation populations in Europe has not been definitively resolved. Remane and Fröhlich (1994) discussed this form (as *Errastunusocellaristatraensis*) based on populations in the Alps but were unsure whether these populations were taxonomically distinct as even a subspecies, or simply ecophenotypic variation. Nickel (2003) indicates specimens from the Bavarian Alps show characters of both forms.

Specimens examined for this project from northwestern North America had variable but generally darker colouration than specimens from eastern North America and low elevations in Europe, although none with the extreme dark forms sometimes seen in high elevation European populations (e.g., Fig. 1E). Slight genitalic differences were also observed, with northwestern specimens typically having shorter subgenital plates (1.38–1.50 times pygofer length, compared to 1.47–1.61, *N* = 5 for each group), more frequent presence of flanges at the base of the aedeagus, and slightly shorter styles.

These differences do not appear to be taxonomically significant. Latitudinal and altitudinal variation in pigmentation (de Oliveira et al. 2004) and genitalic structure (Le Quesne and Woodroffe 1976) are known to occur in other leafhoppers, and these differences do not seem to rise above what might be expected from such variation.

The COI sequences obtained for this study also indicate relatively slight differentiation between these northwestern populations and those elsewhere. Although specimens from northwestern North America and high elevations (1950 m) in the Austrian Alps differ slightly from lower elevation *E.ocellaris*, they are also relatively closely related to a specimen from England which appears to be typical *E.o.ocellaris*.

While resolving the status of *E.ocellaristatraensis* within the European context is not the objective of this study, within the Nearctic region the best treatment appears to be to treat this northwestern population simply as *E.ocellaris*. These populations are considered to be a native element of the fauna, representing the easternmost extent of the species Holarctic distribution.

Hamilton’s (1983) view that populations of *Errastunusocellaris* in the eastern Nearctic represent an introduction from Europe appears to be correct. The earliest specimens of these populations examined are from Hudson Falls, NY (1950, CNC) and Sainte-Flore, QC (1951, CNC). Moore (1944) had earlier recorded this species from Hudson Heights near Montreal, QC beginning in 1942. Although I was not able to examine Moore’s specimens, specimens taken in 1956 from the same locality are all *E.ocellaris* and no *E.sobrinus* have ever been collected from the Ottawa River lowlands, suggesting these are the earliest records of the introduced population. Mapping specimens by collection date (Fig. 8) shows a clear signature of expansion from early records in southern Quebec, eastern Ontario, and northern New York. Montreal is the closest major port to these early records and may represent the point of introduction. COI sequences also provide some support for this population being introduced, with very low divergence between a sequenced specimen from lower elevations in Austria and all eastern Nearctic specimens. Specimens from the Vancouver area (earliest from 1960) likely also represent introductions, although whether these represent a secondary introduction from the East or a separate introduction from the Palearctic is not clear.

The species now occurs commonly in eastern Canada and is easily collected, suggesting that historical records accurately depict its distribution. There are much earlier specimens in the CNC of abundant native species that now often co-occur with *E.ocellaris* in eastern Canada such as *Endriainimicus* (Say) (earliest from Trenton, ON, 1901) and *Diplocolenusevansi* (Ashmead) (earliest from Montreal, QC, 1905), indicating collecting effort that should have yielded specimens of *E.ocellaris* had it been present. In comparison, specimens in the CNC of the native *E.sobrinus* were collected as early as the 1920s, despite its absence from much of southern Ontario and Quebec where early collections were concentrated.

The status of the northwestern population as native or introduced cannot be tested on the same basis. The earliest record of this population is from 1948 (Reindeer Depot, NWT), only slightly predating those from eastern Canada. However, there was very little entomological research in northwestern North America prior to the Northern Insect Survey beginning in 1947 (Freeman 1959), and indeed the first record of *E.sobrinus* in the region is from 1951 (Big Delta, AK). However, this population is mostly likely to be native on the basis of several lines of evidence. First, COI sequences from this population are divergent from the introduced Eastern population, suggesting a different origin. COI haplotypes appearing to originate from this population have also been found in specimens of *E.sobrinus* from as far south as Colorado (see discussion below), and it seems unlikely such introgressed haplotypes could travel so far within a few decades if the population were recently introduced. Finally, the leafhopper fauna in this area is otherwise entirely native, including a number of otherwise Palearctic species restricted in North America to Beringia (Hamilton 1997).

The current extent of distribution for the introduced eastern population is unclear based on the material examined. Collections in the CNC are sparse after approximately 1990 due to reduced collection effort and as the distribution appears to be expanding the current range is probably larger than depicted in the map. A specimen collected in southern Saskatchewan in 2015 (CNC) represents the westernmost confirmed record, excepting the populations around Vancouver. Images of *Errastunus* which may represent *E.ocellaris* are available from online databases and suggest a wider distribution (e.g., from North Carolina https://bugguide.net/node/view/1000457/bgimage and Edmonton, AB https://bugguide.net/node/view/596209/bgimage). However, these records cannot be definitively identified and are not included in the mapped distributions.

### ﻿ Errastunus sobrinus

Taxon classificationAnimaliaHemipteraCicadellidae

(DeLong & Sleesman)

B49D5EDF-0AA2-535C-BEDE-C40E47FE6BD5

Figs 1G, H ; 3 , 5

Latalus ocellaris var. sobrinus DeLong & Sleesman, 1929: 100. Type locality: Slave Lake, AB, Canada.
Deltocephalus sobrinus (DeLong & Sleesman) — Walley 1932 (revised status, comb. nov.).
Errastunus sobrinus (DeLong & Sleesman) — Oman 1949 (comb. nov.).
=Errastunusocellaris (Fallén) — Beirne 1956 (syn. nov.).
Latalus sobrinus (DeLong & Sleesman) — Hamilton 1983 (restored status and comb.).
Latalus (Adarrus) sobrinus (DeLong & Sleesman) — Hamilton 1997 (comb. nov.).

#### Type material.

This species was described from six syntypes from Alberta and an unknown number of syntypes from New York, all females. I was only able to locate two of these, one (labelled as “holotype”) in OSUC and one (labelled as “paratype”) in INHS. There are three specimens in the USNM collected between 1904–1908 in New York; DeLong and Sleesman cite other specimens from USNM in their paper which suggests they could have seen these, but they did not label the specimens or cite specific details so it is not clear if these are syntypes or not.

In order to clarify the application of the name, the specimen in OSUC (OSUC 169881) is here designated lectotype. The labels on this specimen (individual labels separated by /) read: “Slave L., Alta., Aug. 15, 1924, O. Bryant / Grizzly Mt., 3000 ft. / Holotype [red label, handwritten] / Latalusocellaris var sobrinus DeL+S / HOLOTYPE, Errastunussobrinus [red label] / D.M.DeLong Collection / Errastunussobrinus (DeL.+S.)”.

The specimen in INHS (INHS Insect Collection 679931), collected at Slave Lake on Aug 14, and any other former syntypes thus become paralectotypes.

#### Other material examined.

283 specimens (see Supplementary material)

#### Distribution.

Endemic to the Nearctic region, where it has a boreo-montane distribution. Occurs across most of Canada, from Labrador and Nova Scotia in the east to Alaska and British Columbia in the west, and south in the Rocky Mountains to Colorado. Although this species was recorded from Yukon by Hamilton (1997), and likely occurs there based on occurrence in Alaska and British Columbia, all examined *Errastunus* from that territory were *E.ocellaris*. In the east, the southernmost occurrences appear to be associated with higher elevations, with records from the Laurentian, Chic-Choc, Adirondack and White Mountains and the Cape Breton highlands.

#### Host plants.

None of the examined specimens had specific host plants recorded. Unpublished collecting notes from K.G.A. Hamilton suggest this species is associated with grasses in wooded habitats.

#### Remarks.

The combination of morphological, molecular, and distribution data strongly supports the validity of *E.sobrinus* as a distinct species. Morphological differentiation is moderate, but comparable in degree to that found between species in other genera of Paralimnini. The consistent difference in pygofer shape, along with weaker differences in other male genitalic characters, female characters, and external colour all support this interpretation, while the degree of divergence observed between the two clusters of COI sequences is higher than that seen in many morphologically distinct leafhopper species (unpublished data). The generally distinct distributions, with some overlap, also support this variation as specific rather than random variation within a species or geographic differentiation.

The occurrence of some specimens that morphologically appear to belong to *E.sobrinus* but have COI sequences falling in the *E.ocellaris* cluster suggests there has been historic introgression between these species. There is no indication that these specimens are first generation hybrids, as morphologically they appear typical of *E.sobrinus*. All of these specimens were collected within the range of *E.sobrinus*, and outside the known current range of *E.ocellaris*. The sequences of these apparent introgressed individuals fall within the cluster of sequences that includes northwestern North American specimens, suggesting this population is the source of these introgressed haplotypes. The most likely scenario appears to be historic introgression between these northwestern *E.ocellaris* and *E.sobrinus* in an area of overlap, with some haplotypes being retained within populations of *E.sobrinus*.

## ﻿Discussion

Although the two species of *Errastunus* in the Nearctic have mostly distinct ranges, they do overlap both in the east and northwest, and the area of potential overlap is increasing with expansion of the range of adventive populations of *E.ocellaris*.

In the east, although there is broad geographic overlap, actual overlap between the two species may be limited as *E.sobrinus* appears to be absent from most of the lower elevation areas where *E.ocellaris* occurs. One of the few exceptions is at Mt. Washington, NH. The first record seen from that locality is a female of *E.sobrinus* collected in 1929. By 1954, *E.ocellaris* was present, with one collection in 1958 including two male *E.sobrinus* and two female *E.ocellaris*. Recent collecting trips have not yielded either species (Don Chandler, pers. comm.). The few examined collections from the Adirondack Mountains after 1950 were also *E.ocellaris*, while *E.sobrinus* had been present historically based on specimens from Lake Placid (1904, USNM) and Cascade Lake (1908, USNM) (see also Osborn 1922). This may indicate a displacement of *E.sobrinus*, although more thorough collecting effort is needed to determine the current status in these areas.

Geographic overlap between northwestern populations of the two species is also extensive, although there are no known cases where both species have been collected at a single locality. It is possible there is some differentiation in habitat preference that keeps the species apart. However, given the apparent mitochondrial introgression observed in *E.sobrinus* there has clearly been at least historic contact between these populations.

Given the fairly deep divergence between the species and their respective distributions, it seems likely that the two species speciated while on separate continents, with *E.ocellaris* in Eurasia and *E.sobrinus* in North America. A population of *E.ocellaris* may have then entered North America via the Beringian land bridge and hybridized with *E.sobrinus* either within Beringia or after coming into contact during later glacial retreat. There is no evidence of ongoing introgression in current areas of overlap or mitochondrial introgression from *E.sobrinus* into *E.ocellaris*, but more sequencing effort in these areas would be needed to test this thoroughly.

## Supplementary Material

XML Treatment for Errastunus (Errastunus)

XML Treatment for Errastunus ocellaris

XML Treatment for Errastunus sobrinus

## ﻿Additional information

### Author ORCIDs

Joel H. Kits https://orcid.org/0000-0003-2685-0567

### Data availability

All of the data that support the findings of this study are available in the main text or Supplementary Information.

## ﻿Appendix 1

**Works using *Cicadaocellaris* Fallén as a valid name**

1. Bornholdt G (2002) Untersuchungen zum Einfluss von Düngung und Nutzungsaufgabe auf die Zikadenfauna von Borstgrasrasen und Goldhaferwiesen. Beiträge zur Zikadenkunde 5: 14–26.
2. Dmitriev DA (1999) A new subgenus of the genus *Adarrus* Ribaut, 1947 (Homoptera: Cicadellidae). Zoosystematica Rossica 8: 79–82.
3. Dmitriev DA (2001) A new subgenus of *Errastunus* Rib. for *Adarrusdaedaleus* Logvinenko, 1966 with a new record from Russia (Homoptera: Cicadellidae). Zoosystematica Rossica 9: 351–352.
4. Hamilton KGA (1983) Introduced and native leafhoppers common to the Old and New Worlds (Rhynchota: Homoptera: Cicadellidae). The Canadian Entomologist 115: 473–511. https://doi.org/10.4039/Ent115473-5
5. Hamilton KGA (1997) Leafhoppers (Homoptera: Cicadellidae) of the Yukon: dispersal and endemism. In: Danks HV, Downes JA (Eds) Insects of the Yukon. Biological Survey of Canada (Terrestrial Arthropods), Ottawa, pp 337–375.
6. Heller FR (1975) *Adarrusocellaris* (Fall.) ssp. *tatraensis* ssp. nova. (Homoptera, Cicadellidae). Stuttgarter Beiträge zur Naturkunde A 288: 1–3.
7. Klejdysz T, Klukowski Z, Pruszyński G, Kubasik W (2018) New data and a checklist of Dryinidae (Hymenoptera) from Poland, and their role in controlling leafhopper and planthopper crop pests (Hemiptera: Cicadomorpha, Fulgoromorpha). Polish Journal of Entomology 87: 41. https://doi.org/10.2478/pjen-2018-0003
8. Kobiałka M, Michalik A, Szwedo J, Szklarzewicz T (2018) Diversity of symbiotic microbiota in Deltocephalinae leafhoppers (Insecta, Hemiptera, Cicadellidae). Arthropod Structure & Development 47: 268–278. https://doi.org/10.1016/j.asd.2018.03.005
9. Kruess A, Tscharntke T (2002) Contrasting responses of plant and insect diversity to variation in grazing intensity. Biological Conservation 106: 293–302. https://doi.org/10.1016/S0006-3207(01)00255-5
10. Morris MG (1981) Responses of grassland invertebrates to management by cutting: IV Positive responses of Auchenorhyncha. Journal of Applied Ecology 18: 763–771. https://doi.org/10.2307/2402368
11. Nast J (1987) The Auchenorrhyncha (Homoptera) of Europe. Annales Zoologici 40: 536–661.
12. Nickel H (2003) The leafhoppers and planthoppers of Germany (Hemiptera, Auchenorrhyncha): patterns and strategies in a highly diverse group of phytophagous insects. Pensoft Publishers, Sofia-Moscow, 460 pp.
13. Nickel H, Hildebrandt J (2003) Auchenorrhyncha communities as indicators of disturbance in grasslands (Insecta, Hemiptera)—a case study from the Elbe flood plains (northern Germany). Agriculture, Ecosystems & Environment 98: 183–199. https://doi.org/10.1016/S0167-8809(03)00080-X
14. Nickel H, Remane R (2002) Artenliste der Zikaden Deutschlands, mit Angabe von Nährpflanzen, Nahrungsbreite, Lebenszyklus, Areal und Gefährdung (Hemiptera, Fulgoromorpha et Cicadomorpha). Beiträge zur Zikadenkunde 5: 27–64.
15. Novikov DV, Novikova NV, Anufriev GA, Dietrich CH (2006) Auchenorrhyncha (Hemiptera) of Kyrzgyz grasslands. Russian Entomology Journal 15: 303–310.
16. Ossiannilsson F (1983) The Auchenorrhyncha (Homoptera) of Fennoscandia and Denmark, Volume 3. Family Cicadellidae: Deltocephalinae, Catalogue, Literature and Index. Fauna Entomologica Scandinavica 7: 315 pp. https://doi.org/10.1163/9789004273320
17. Prestidge RA (1982) The influence of nitrogenous fertilizer on the grassland Auchenorrhyncha (Homoptera). Journal of Applied Ecology 19: 735–749. https://doi.org/10.2307/2403278
18. Remane R, Fröhlich W (1994) Beiträge zur Chorologie einiger Zikaden–Arten (HomopteraAuchenorrhyncha) in der Westpaläarktis. Marburger Entomologische Publikationen 2: 131–188.
19. Reynolds DR, Chapman JW, Stewart AJ (2017) Windborne migration of Auchenorrhyncha (Hemiptera) over Britain. European Journal of Entomology 114: 554–564. https://doi.org/10.14411/eje.2017.070
20. Riedle‐Bauer M, Sára A, Regner F (2008) Transmission of a stolbur phytoplasma by the Agalliinae leafhopper *Anaceratagalliaribauti* (Hemiptera, Auchenorrhyncha, Cicadellidae). Journal of Phytopathology 156: 687–690. https://doi.org/10.1111/j.1439-0434.2008.01416.x
21. Schlosser L, Holzinger WE (2017) Zur Zikadenfauna (Hemiptera: Auchenorrhyncha) der Lafnitzwiesen bei Wörth (Steiermark, Österreich). Cicadina 17: 53–61.
22. Söderman G, Gillerfors G, Endrestöl A (2009) An annotated catalogue of the Auchenorrhyncha of Northern Europe:(Insecta, Hemiptera: Fulgoromorpha et Cicadomorpha). Cicadina 10: 33–69.
23. Świerczewski D (2007) A food plant study of the Auchenorrhyncha of the Częstochowa upland, southern Poland (Insecta, Hemiptera). Beiträge zur Zikadenkunde 9: 15–22.
24. Thompson P (1978) The oviposition sites of five leafhopper species (Horn. Auchenorrhyncha) on *Holcusmollis* and *H.lanatus*. Ecological Entomology 3: 231–240. https://doi.org/10.1111/j.1365-2311.1978.tb00923.x
25. Waloff N, Thompson P (1980) Census data of populations of some leafhoppers (Auchenorrhyncha, Homoptera) of acidic grassland. Journal of Animal Ecology 49: 395–416. https://doi.org/10.2307/4254

## Supplementary materials

Supplementary material 1 Data for examined *Errastunus* specimens Data type occurences Explanation note CSV file (Darwin Core compatible) with collection and identification data for museum specimens examined for this study. https://binary.pensoft.net/file/901466This dataset is made available under the Open Database License (http://opendatacommons.org/licenses/odbl/1.0/). The Open Database License (ODbL) is a license agreement intended to allow users to freely share, modify, and use this Dataset while maintaining this same freedom for others, provided that the original source and author(s) are credited.Joel H. Kits
